# Histidine Protonation and Conformational Switching in Diphtheria Toxin Translocation Domain

**DOI:** 10.3390/toxins15070410

**Published:** 2023-06-25

**Authors:** Mykola V. Rodnin, Victor Vasques-Montes, Alexander Kyrychenko, Nuno F. B. Oliveira, Maithri M. Kashipathy, Kevin P. Battaile, Justin Douglas, Scott Lovell, Miguel Machuqueiro, Alexey S. Ladokhin

**Affiliations:** 1 Department of Biochemistry and Molecular Biology, University of Kansas School of Medicine, Kansas City, KS 66160, USAa.v.kyrychenko@karazin.ua (A.K.); 2 Institute of Chemistry and School of Chemistry, V. N. Karazin Kharkiv National University, 61022 Kharkiv, Ukraine; 3 Institute of Biosystems and Integrative Sciences, University of Lisbon, 1749-016 Lisbon, Portugalmachuque@ciencias.ulisboa.pt (M.M.); 4 Protein Structure and X-ray Crystallography Laboratory, University of Kansas, Lawrence, KS 66047, USAswlovell@ku.edu (S.L.); 5 NYX, New York Structural Biology Center, Upton, NY 10027, USA; 6 COBRE Bio-NMR Laboratory, University of Kansas, Lawrence, KS 66045, USA; justindouglas@ku.edu

**Keywords:** diphtheria toxin structure, NMR spectroscopy, mutations of histidines, X-ray crystallography, constant-pH simulations, acidification, conformational switching

## Abstract

Protonation of key histidine residues has been long implicated in the acid-mediated cellular action of the diphtheria toxin translocation (T-) domain, responsible for the delivery of the catalytic domain into the cell. Here, we use a combination of computational (constant-pH Molecular Dynamics simulations) and experimental (NMR, circular dichroism, and fluorescence spectroscopy along with the X-ray crystallography) approaches to characterize the initial stages of conformational change happening in solution in the wild-type T-domain and in the H223Q/H257Q double mutant. This replacement suppresses the acid-induced transition, resulting in the retention of a more stable protein structure in solutions at pH 5.5 and, consequently, in reduced membrane-disrupting activity. Here, for the first time, we report the pK_a_ values of the histidine residues of the T-domain, measured by NMR-monitored pH titrations. Most peaks in the histidine side chain spectral region are titrated with pK_a_s ranging from 6.2 to 6.8. However, the two most up-field peaks display little change down to pH 6, which is a limiting pH for this protein in solution at concentrations required for NMR. These peaks are absent in the double mutant, suggesting they belong to H223 and H257. The constant-pH simulations indicate that for the T-domain in solution, the pK_a_ values for histidine residues range from 3.0 to 6.5, with those most difficult to protonate being H251 and H257. Taken together, our experimental and computational data demonstrate that previously suggested cooperative protonation of all six histidines in the T-domain does not occur.

## 1. Introduction

**Figure 1:**
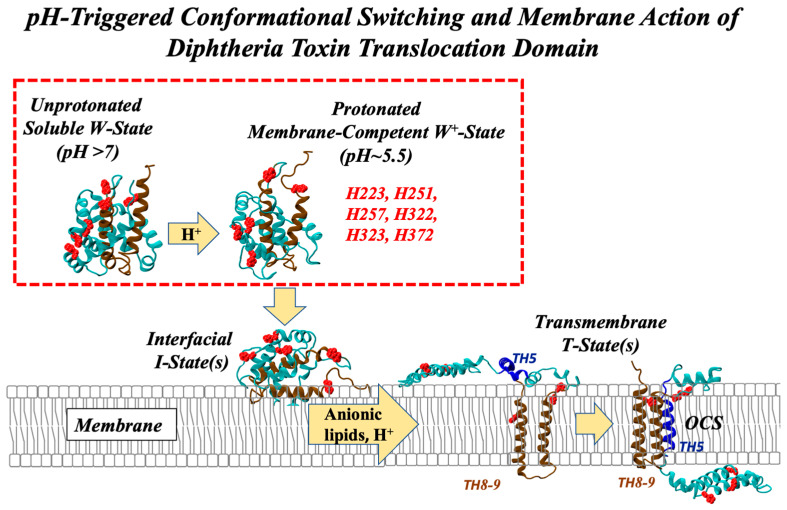
General scheme of acid-induced conformational switching and membrane insertion of Diphtheria Toxin T-domain (for details see [21,22]). The insertion pathway is initiated by the conversion of the water-soluble unprotonated W-state into the protonated membrane-competent state W^+^-state and its subsequent binding to the membrane interface. The conversion from a family of interfacial states into two predominant transmembrane states, with different topologies of the N-terminus, is facilitated by the presence of anionic lipids. The exact molecular mechanism of the translocation of the Catalytic domain of the toxin, attached to the T-domain’s N-terminus, remains unknown. The evidence from mutagenesis indicates that the formation of the so-called Open-Channel State, OCS (illustrated by the cartoon on the right) is not necessary for the translocation and that the OCS does not constitute the translocation pathway [23] (the six native histidines are listed in the scheme and are highlighted in red. The consensus insertion hairpin formed by helices TH8 and TH9 is highlighted in brown. Helix TH5, which can have either interfacial or transmembrane topology, is highlighted in blue in the two membrane-inserted conformations. No high-resolution structures are available for the T-domain in the lipid bilayer, and the presented schemes, developed from various spectroscopic and computational experiments, are shown for illustration purposes only). The focus of this study is on the initial conformational transition occurring in solution (red ractangle), which is associated with the protonation of the histidine residues.

The action of many pore-forming bacterial toxins [1,2,3,4,5] and colicins [6,7] involves the initial conversion of a protein structure from a water-soluble to a membrane-inserted form. The latter transition constitutes one of the least understood cellular processes and is shared with many other cellular systems (e.g., tail-anchor proteins [8,9] and multiple proteins of the Bcl-2 family of apoptotic regulators [10,11,12]). Our long-term objective is to describe at the molecular level the mechanisms of pH-triggered conformational switching of the diphtheria toxin T-domain, which serves as a model for membrane insertion/translocation transitions. The function of the T-domain is to translocate the catalytic domain across the lipid bilayer in response to the acidification of the endosome [13]. Remarkably, such a complex task is performed by this small 180-residue protein without the help of any additional translocation machinery. Despite the efforts from many labs [14,15,16,17,18,19,20], the exact mechanism of translocation remains unknown. It is clear, however, that the central issue is a membrane-mediated refolding process, encompassing targeting the membrane, and a series of interface-directed conformational changes, resulting in multiple charged groups crossing the bilayer and populating several transmembrane (TM) conformations that span the entire bilayer (Figure 1).

In this study, we focus on the first step of the acid-induced conformational transition of the diphtheria toxin T-domain, namely the formation of the protonated membrane-competent W^+^-state in solution (Figure 1, dashed red rectangle). In our previous studies, we have used mutagenesis to demonstrate that the replacement of histidine residues modulates the efficiency and the pH dependence of this transition [21]. Specifically, we suggested that contrary to the views expressed in the literature [24], the protonation of the six native histidines does not occur cooperatively and that individual histidines have distinct roles in modulating various stages of the insertion pathway. Specifically, we postulated an interplay in the protonation of H223 and H257 [25,26] and explored the properties of single replacements of these residues. Here, we extend our study to the H223Q/H257Q T-domain Double Mutant (DM) and demonstrate its suppressed ability to undergo the necessary conformational switching at acidic pH. Additionally, for the first time, we have directly demonstrated the protonation of the individual histidines in the T-domain using NMR spectroscopy and constant-pH molecular dynamics (MD) simulations.

## 2. Results and Discussion

**Figure 2:**
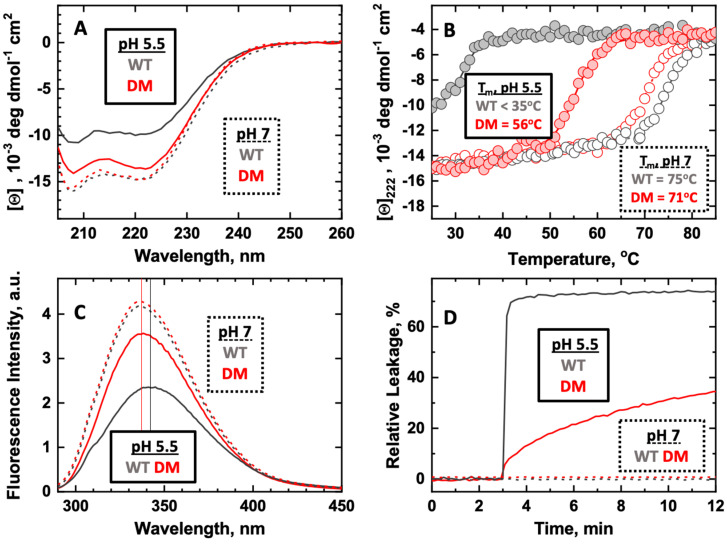
Comparison of pH-induced conformational switching (**A**–**C**) and membrane action (**D**) of the T-domain wild type (WT, *black lines*) and H223Q/H257Q Double Mutant (DM, *red lines*). Dashed curves and open symbols correspond to pH 7, and solid curves and symbols to pH 5.5. The replacement of the two histidines with non-protonatable residues suppresses conformational change leading to the formation of the membrane-competent W^+^-state, as seen by the reduced changes in CD (**A**) and tryptophan fluorescence spectra (**C**). The thermal stability of the helical structure, as measured by changes of ellipticity at 222 nm, is much higher at pH 5.5 for the DM than that for the WT T-domain (**B**). Subsequently, the ability of the DM to induce the leakage of the membrane content at pH 5.5 is much reduced compared to the WT (**D**).

Formation of the membrane-competent W+-state occurs in solution upon acidification of the environment to pH below 6 and is accompanied by structural destabilization and partial unfolding of the T-domain [27]. The latter manifests itself in changes in helical content (Figure 2A), a decrease in thermal stability (Figure 2B), and partial exposure of two native tryptophan residues. In the case of the WT, a red-shift of the maximum of the emission spectrum is observed at a low pH value, which indicates exposure to a more hydrophilic environment, which also could cause the observed decrease in quantum yield (Figure 2C). As demonstrated in Figure 2A–C, the CD, thermal stability, and tryptophan fluorescence of the H223Q/H257Q double mutant (DM) of the T-domain (*red*) changes less upon acidification from pH 7 (*dashed/hollow*) to 5.5 (*solid/filled*) than the WT (*grey*). These results suggest the inhibited formation of the membrane-competent conformation at pH 5.5 for the DM compared to the WT. Consequently, the ability of the DM to release the content of vesicles under these conditions is substantially reduced (Figure 2D).

In order to further analyze the putative pH-dependent structuring of the TH2-TH3 region, we crystallized a full-length form of diphtheria toxin (DT) harboring the H223Q/H257Q mutations. Notably, two crystal forms were obtained from crystallization solutions at pH 5.5 and 4.5. The first crystal form (DT-DM-A) was isomorphous with the aforementioned WT structures, whereas the other form (DT-DM-B) belonged to another crystal form (Table 1). 

**Figure 3:**
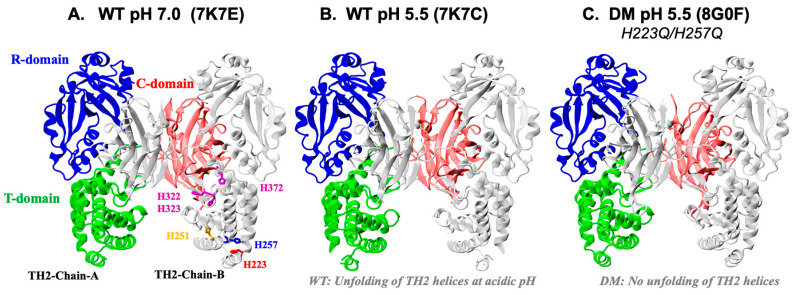
Comparison of the high-resolution crystallographic structures of the Diphtheria Toxin dimers: WT at pH 7 (**A**), 5.5 (**B**), and DM at pH 5.5 (**C**). In each case, the toxin forms a domain-swapped dimer shown, with one chain colored uniformly in grey and the other colored in blue (Receptor-binding domain), green (Translocation domain), and red (Catalytic domain). The six histidines are shown only for the former chain in panel A and are colored the same way as their corresponding labels. The first two structures, published in our previous study [34], reveal acid-induced loss of the helical structure of TH2 of the WT T-domain (labeled panel A as TH2-A and TH2-B for each monomer). This change is not observed when H223 and H257 are replaced in the DM of both crystal forms (PDB 8G0F and 8G0G) (C). Otherwise, the overall fold is not affected by the replacement of the two histidines (local rearrangements are shown in Figure 4).

Not surprisingly, the mutant structures also form domain-swapped dimers and are similar to the WT structures. Superposition onto the WT DT obtained at pH 5.0 (7K7B) yielded RMSD deviations of 0.57 Å (999 residues, DT-DM-A) and 0.62 Å (999 residues, DT-DM-B). In addition, the DT-DM structures were similar to the WT structure obtained at pH 7.0 (7K7E), and superpositions yielded RMSD deviations of 0.41 Å (1000 residues) (the overall similarity in the environments of titratable residues in the WT and DM T-domain are also highlighted by the results of the simplified pK_a_ calculations presented in the Supplemental Table S1. Note that those are only used for comparison of the two proteins, and a rigorous analysis of pK_a_ values in the WT with the help of Constant-pH MD simulations is presented below). Interestingly, the TH2 helices in the mutant structures are well ordered in a similar manner as observed for the WT pH 7.0 structure (Figure 3A–C) and could be modeled on the electron density maps. 

**Figure 4:**
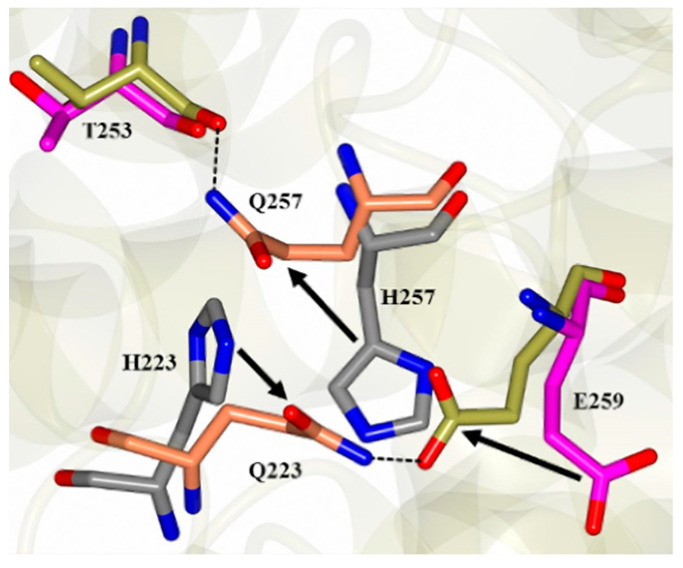
The local rearrangement of the packing around residues 223 and 257 in the DM vs. the WT T-domain. Q223 in DM (8G0G, gold/orange) forms a hydrogen bond with E259. This results in the movement of residue Q257 relative to the WT structure (7K7E, magenta/gray), and a new sidechain interaction is formed with T253.

This suggests that the mutations serve to stabilize the TH2 helices regardless of pH. Interestingly, the Q223/Q257 sidechain conformations are distinctly different from the H223/H257 residues in the WT structures. Concerted movement of Q223, Q257, and E259 in DT-DM-B results in the formation of new hydrogen bond interactions, as shown in Figure 4. Moreover, the acid-induced destabilization of the T-domain’s short helix TH2, previously reported for the WT [34], is no longer observed with the DM (Figure 3C). The latter is overall consistent with the spectroscopic evidence presented above in Figure 2 for the lack of efficient conversion of the membrane-competent state in the mutant. 

**Figure 5:**
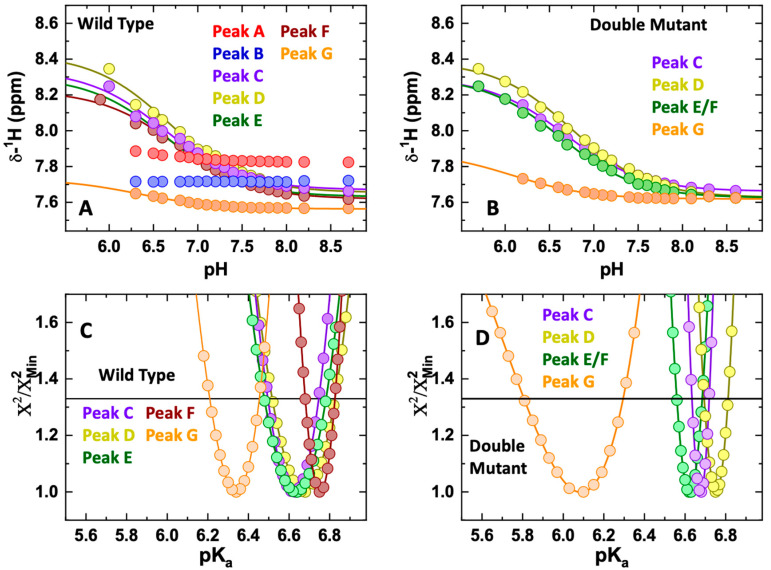
pH-dependent changes in the histidine side chain NMR peaks in the T-domain WT (**A**) and DM (**B**). The two WT peaks (panel A) that show no change (Peak A and Peak B) are likely to belong to H223 and H257, as the corresponding peaks are absent in the DM (panel B). The rest of the peaks appear to have very similar patterns in DM and WT (note that the number of the peaks in the region between 8.3 and 7.5 ppm is greater than the number of histidine side chains in both the WT and DM construct. This discrepancy is attributed to either dynamic conformational heterogeneity or overlap from other side chain resonances. Panels C and D represent the support-plane analysis of the titratable peaks in the WT (panel C) and the DM T-domain (panel D), which provides a measure of the reliability of pKa determination. The minimum of the curve corresponds to the most probable pKa, while the intercepts with the solid line at 1.33 corresponds to a range of one standard deviation. Even for the most visually different curves corresponding to the Peak G, the resulting confidence intervals of pKa overlap for the WT ((**C**), pKa = [6.2–6.5]) and the DM ((**D**) pKa = [5.8–6.3]).

We examined the protonation states of histidine residues in the T-domain WT and DM using 1D ^1^H NMR spectroscopy. Even in the absence of a complete resonance assignment, the resulting pH-dependent chemical shifts of the WT (Figure 5A) clearly indicate that protonation of various histidines occurs at different pH values. To guide the discussion, we have labeled each peak in the region between 8.3 and 7.5 ppm with the letters “A” to “G” for the most to least downfield under basic conditions. Although the peaks broaden as the pH decreases, indicating aggregation, it is facile to track the pH-dependence of each individual resonance stepwise down to pH 6.3. Note that since NMR experiments require higher protein concentrations than fluorescence and CD spectroscopy, significant precipitation occurs at pH 6. The peaks “A” and “B” are not changed in the experimentally accessible pH range. Moreover, neither peaks “A” nor “B” are present in the DM data (Figure 5B), leading us to assign these resonances to H223 and H257. The pK_a_ values of the remaining peaks were accessed by non-linear least square fitting to Equation (1) and subjected to the support-plane analyses presented in Figure 5C,D for the WT and DM T-domain, respectively. The replacement of the two histidines with glutamines in the DM has little effect on the pK_a_s of the other histidine residues, again indicating an absence of a cooperative transition that would result in the protonation of all histidine residues.

**Figure 6:**
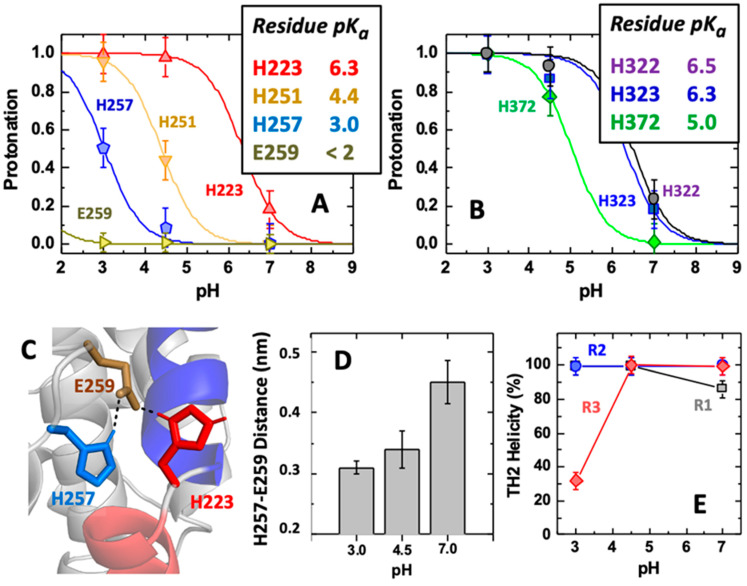
Summary of the constant-pH MD simulations of the WT T-domain. The individual pH titration curves and calculated pK_a_ values for residues H223, H251, H257, and E259 (**A**) and H322, H323, and H372 (**B**). Structure representation of DTT after 150 ns at pH 4.5 (**C**). The protein is shown in the cartoon (*light gray*) with TH1 highlighted in blue and TH2 in red color, and with residues H223 (*red*), H257 (*blue*), and Glu259 (*olive*) represented as sticks. The average distances between H257 and E259 at different pH values illustrate the acidic residue sequestering induced by protonation (**D**). The average helicity content of TH2, at different pH values at three different replicates, highlights the significant loss of structure in one replicate (R3) induced by H257 protonation (**E**).

Constant-pH Molecular Dynamics (CpHMD) simulations are known to provide a detailed analysis of the coupling between the protonation of key amino acid residues and the conformational transitions that they may trigger [35,36]. In our work, we used this approach to study the wild-type form of the T-domain (Figure 6A,B) at the following pH values: 7.0, 4.5, and 3.0. These values correspond to the cases of (a) pre-endosomal acidification, (b) acidified late endosomes, and (c) a low pH non-physiological control, respectively. The use of the latter low pH value aims at increasing the protonation sampling of the key residues, which should accelerate the larger conformational transitions that are often very slow and rare in the timescale of our computer simulations (150 ns). Our simulations (Figure 6A) show that H223 has an unperturbed pK_a_ value of 6.3 (here we use the term “unperturbed” for the standard state of histidine amino acid sidechain in solution in the absence of the protein moiety), which is expected due to the significant solvent exposure observed during our simulations. H251 is facing a different region, and since it is partially buried, its pK_a_ value is decreased to 4.4. In contrast, H257 is located near H223 and shows a strongly shifted pK_a_ value of 3.0, indicating a significant overstabilization of its neutral form. This is usually explained by desolvation effects and/or the interaction with another cationic residue (e.g., H223 in our case; Figure 6C). Thus, in this scenario, the protonation of H257 will require at least a transient stabilization from an acidic residue. 

We suggest that this role is played by E259, which in our simulations appears to be always ionized under tested conditions with pK_a_ < 2. Thus, E259 can be sequestered at lower pH values to establish an electrostatic triad with H223 and H257 (Figure 6D). We have noticed that in one of the three replica runs (R3), the protonation of H257 appears to be accompanied by a local destabilization of the TH2 helix (Figure 6E), contributing to the water exposure of the H223/E259/H257 electrostatic triad. Overall, this coupling between H257 protonation and structural reorganization may indeed be the initial trigger that leverages the larger conformational transition associated with membrane insertion in the endosomes. In addition, the latter can also be influenced by the cluster of three already protonated C-terminal histidines (H322, H323, H372), known to play a role in the later stages of the insertion pathway [37,38].

## 3. Conclusions and Perspectives

The data presented here clearly indicate that protonation of the key histidine residues involved in the conformational switching in the diphtheria toxin T-domain occurs at a wide range of pH values. The replacement of H223 and H257 with non-titratable Q residues suppresses the ability of the T-domain to form a membrane-competent state and disrupt the bilayer yet has little effect on the protonation of the other four histidines. The three C-terminal histidines, H322, H323, and H372, appear to be protonated before and independently from the formation of the membrane-competent conformation. We suggest that the protonation coupling between H223 and H257 involves their interaction with a neighboring residue E259, which remains negatively charged up to very low pH values. The coupling within the H223/H257/E259 triad and the role of H251 will be the focus of further investigations. 

## 4. Materials and Methods

### 4.1. Materials 

Phospholipids used in this study: palmitoyl-oleoyl-phosphatidylcholine (POPC) and palmitoyl-oleoyl-phosphatidylglycerol (POPG) were purchased from Avanti Polar Lipids (Alabaster, AL, USA). The fluorescent dye 8-aminonaphthalene-1,3,6-trisulfonic acid, disodium salt (ANTS), and quencher p-xylene-bis-pyridinium bromide (DPX) were obtained from Invitrogen (Carlsbad, CA, USA).

### 4.2. Preparation of the T-Domain and Full-Length DT 

Both full-length DT and the T-domain were prepared as described in [25]. The E148S/C201S mutant with strongly reduced catalytic activity and cytotoxicity [39] was used as a template for the expression of a full-length toxin.

### 4.3. Tryptophan Fluorescence 

Fluorescence was measured using SPEX Flurolog FL 3–22 steady-state fluorescence spectrometer (Jobin Yvon, Edison, NJ, USA) equipped with double grating excitation and emission monochromators as described in [25,26]. The measurements were carried out at 25 °C in 2 × 10 mm cuvettes oriented perpendicular to the excitation beam. For tryptophan fluorescence measurement, the excitation-emission wavelength was 280 nm, and emission spectra were recorded between 290 nm and 450 nm using excitation and emission spectral slits of 2 and 4 nm, respectively. Normally, we mixed the sample (taken from a concentrated stock) with LUV maintaining the T domain and lipid concentrations at 1 µM and 1 mM, respectively, and rapid acidification was achieved by the addition of small amounts of 2.5 M acetic buffer. All spectra were recorded after 30 min of incubation to ensure the equilibration of the sample. 

### 4.4. CD Measurement and Analysis of Thermal Unfolding

CD spectra and thermal unfolding curves were recorded using an upgraded Jasco-720 spectropolarimeter (Japan Spectroscopic Company, Tokyo, Japan), as described in [25,26]. The thermal unfolding was analyzed using thermodynamic equations for a reversible two-state, N-to-U unfolding transition [40].

### 4.5. Vesicle Preparation and Leakage Assay

Large Unilamellar Vesicles (LUV) were prepared by extrusion [41,42], and their permeabilization was studied fluorometrically by following the release of ANTS dye co-encapsulated with the DPX quencher [43]. A mixture of POPC and POPG (1:1, molar ratio) in chloroform was dried under the flux of nitrogen and then dried overnight under a high vacuum. The phospholipid film was suspended in 50 mM phosphate buffer, pH 8.0 containing 1 mM ANTS and 10 mM DPX and extruded 10 times through 100 nm Nucleopore polycarbonate membranes (Millipore, St. Louis, MO, USA). Each extrusion was followed by freezing in liquid nitrogen and thawing. Loaded vesicles were isolated from non-included components by size-exclusion chromatography on a 1 × 30 cm Superose 6 column. Fluorescence was recorded on SPEX Fluorolog FL3-22 steady-state fluorescence spectrometer (Jobin Yvon, Edison, NJ, USA) using a 2 × 10 mm cuvette oriented perpendicular to the excitation beam. Excitation and emission wavelengths were 353 and 520 nm, respectively. The permeabilization reaction was completed by the addition of 20 µL 20% Triton-X100, allowing the determination of the fluorescence signal associated with 100% content release.

### 4.6. NMR Measurements

All NMR data were acquired at 308 K on a Bruker AV 800 MHz NMR (Bruker, Billerica, MA, USA) spectrometer equipped with a triple resonance inverse cryoprobe. The samples were transferred to a 5 mm NMR tube (Wilmad Lab-glass, Vineland, NJ, USA). pH was adjusted by adding small aliquots (ca. 5 μL) of dilute HCl (ca. 5 mM) and monitored using a calibrated NMR tube pH electrode (Wilmad Lab-glass, Vineland, NJ). Typical parameters for the NMR experiment are the following: pulse program zgesgp, interscan delay 2 s, pulse power 35 kHz, number of scans 64. All data were acquired and processed using Bruker Topspin software.

### 4.7. Titration Analysis

The pH-dependent data were fitted with the following equation to obtain the pK_a_ values:

(1) $$I=\frac{I_{N}+I_{L}(10^{m\left({pK}_{a}-pH\right)})}{1+10^{m({pK}_{a}-pH)}}$$

where *I* is the protonation-associated signal at a particular pH, I*_N_* and I*_L_* are the limiting values at high and low pH, respectively, and m is the transition slope. For NMR titrations, the position of the spectral shift was taken as *I*. For Constant-pH MD simulation, *I* was taken to be the actual population of protonated specie, I*_N_* = 0, I*_L_* = 1, and *m* = 1.

### 4.8. Support Plane Analysis

The robustness of the determined pK_a_ parameters was determined by subjecting the pH-dependent fits of NMR spectral shifts to support plane analysis [44]. Briefly, a series of least-square fits were generated with Equation (1), in which the pK_a_ parameter was fixed at different values with small steps around the most optimal solution. The ratio between every X^2^ value and the optimal X^2^ solution (X^2^_Min_) was plotted, and a cut-off corresponding to a single standard deviation (X^2^/X^2^_Min_ = 1.33) was used to identify the range of pK_a_ values.

### 4.9. Computational Methods

The X-ray crystallographic structure (PDB: 1F0L) [45] was used to set the MD simulations of the T-domain. The system was inserted into a dodecahedric box and solvated with 8404 SPC water molecules [46]. MD simulations were performed using GROMACS 5.1.5 software package [47] and with the GROMOS 54A7 force field [48,49,50]. In the CpHMD method [35,36], the Poisson–Boltzmann calculations were performed with Delphi v5.1 [51] using a dielectric constant of 2 and 80 for solute and solvent, respectively, and an ionic strength of 0.1 M. The molecular surface was generated with a 1.4 Å probe and an exclusion layer of 2 Å. Two grids of 1 Å (large) and 0.25 Å (focusing) were used to calculate the electrostatic potential with a 0.01 convergence threshold and relaxation values of 0.75 for both linear and non-linear iteration steps. The PETIT v1.6 software [52] was used to perform 100 k Monte Carlo cycles to sample the protonation states from the PB-derived energies.

In all MD simulations, the temperature was kept at 310 K using the v-rescale thermostat [53] with a coupling constant of 0.1 ps. The Parrinello–Rahman barostat [54] was used to maintain the isotropic pressure constant at 1 bar with the compressibility of 4.5 × 10^−5^ bar^−1^ and coupling constant of 1 ps. Long-range interactions were treated with the atomistic Verlet scheme and a 1.4 nm single cutoff. The van der Waals interactions were truncated after the cut-off, while the electrostatic interactions were treated with particle-mesh-Ewald (PME) [55]. The protein bonds were constrained using the P-LINCS algorithm [56] for the protein, while water molecules were constrained with SETTLE [57]. The system energy minimization was performed with the steepest descent algorithm for 10 k steps without constraints, followed by ~100 steps with all bonds constrained. The temperature was initialized in a 100 ps short MD simulation, followed by the initialization of pressure in another segment of 200 ps.

Three replicates of the initialized protein were simulated with our CpHMD method at three different pH values, 3.0, 4.5, and 7.0, for 150 ns. All aspartate, glutamate, and histidine residues in the protein were allowed to titrate. To keep the system near charge neutrality, a requirement of using PME, we performed a pre-run of 10 ns for each pH value and estimated the number of counter-ions required. For pH 3.0 and 4.5, a total of 13 and 2 Cl^–^ ions were added, respectively. At pH 7, due to a negative charge in the protein, 10 Na^+^ ions were added to achieve the required charge neutralization.

### 4.10. Crystallization and Data Collection

Purified diphtheria toxin (DT) constructs were concentrated at 19.1 mg/mL (H223Q/H257Q double mutant, DM) in 50 mM disodium phosphate pH 8.0 for crystallization screening. All crystallization experiments were set up using an NT8 drop setting robot (Formulatrix Inc.) and UVXPO MRC (Molecular Dimensions) sitting drop vapor diffusion plates at 18 °C. Additionally, 100 nL of protein and 100 nL of crystallization solution were dispensed and equilibrated against 50 uL of the latter. Crystallization was reproduced in CombiClover 500 (Rigaku Reagents) sitting drop plates. Crystals of the double mutant (DM) form of DT (H223Q/H257Q) were obtained from two conditions as follows. DT-DM-A: Berkeley screen (Rigaku Reagents) condition E6 (20% (*w*/*v*) PEG 2000 MME, 100 mM sodium citrate tribasic pH 5.5, 200 mM sodium malonate pH 5.0), and DT-DM-B: Proplex HT screen (Molecular dimensions) condition C2 (20% (*w*/*v*) PEG 4000, 100 mM sodium citrate pH 4.5). Crystals were transferred to a cryoprotectant solution composed of 80% crystallization solution and 20% (*v*/*v*) PEG 200, harvested with a cryoloop, and stored in liquid nitrogen. X-ray diffraction data were collected at the Advanced Photon Source beamline 17-ID using a Dectris Pilatus 6M pixel array detector. 

### 4.11. Structure Solution and Refinement

Intensities were integrated using XDS [58,59] via Autoproc [60], and the Laue class analysis and data scaling were performed with Aimless [28], which indicated that the crystals belonged to the triclinic space group *P*1 and DT-DM-A and DT-DM-B adopted different crystal forms. The Matthews coefficient [61] for all data indicated that a non-crystallographic dimer was present in the asymmetric unit. The structure solution was conducted by molecular replacement with Phaser [62] using a previously determined DT structure as the search model (PDB 7K7B). Model refinement and manual model building were conducted with Phenix and Coot [63], respectively. Disordered sidechains were truncated to the point for which electron density could be observed. Structure validation was conducted with Molprobity [64], and figures were prepared using the CCP4MG package [65]. Structure superposition was carried out with GESAMT [66]. Crystallographic data are provided in Table 1.

### 4.12. Depositions

Coordinates and structure factors were deposited to the Worldwide Protein Databank (wwPDB) with the accession codes 8G0F (DT-DM-A) and 8G0G (DT-DM-B).

## Figures and Tables

**Table 1:** Crystallographic data for diphtheria toxin structures.

Structure PDB Code	DT-DM-A 8G0F	DT-DM-B 8G0G
Data Collection		
Unit-cell parameters (Å, °)	a = 69.55, b = 69.47, c = 72.88, α = 118.0, β = 93.9, γ = 99.3	a = 69.36, b = 69.40, c = 69.67, α = 64.5, β = 76.5, γ = 81.2
Space group	*P*1	*P*1
Resolution (Å) ^1^	46.78–2.25	49.57–2.10
Wavelength (Å)	1.0000	1.0000
Temperature (K)	100	100
Observed reflections	182,683	217,428
Unique reflections	52,984	63,799
<I/σ(I)> ^1^	9.7 (1.7)	9.7 (1.9)
Completeness (%) ^1^	95.8 (96.7)	96.1 (96.9)
Multiplicity ^1^	3.4 (3.4)	3.4 (3.4)
*R*_merge_ (%) ^1,2^	7.4 (72.7)	6.8 (65.0)
*R*_meas_ (%) ^1,4^	8.8 (86.9)	8.1 (77.6)
*R*_pim_ (%) ^1,4^	4.7 (46.9)	4.3 (41.8)
CC_1/2_ ^1,5^	0.997 (0.692)	0.997 (0.703)
**Refinement**		
Resolution (Å) ^1^	36.31–2.25	37.00–2.10
Reflections (working/test) ^1^	50,351/2608	60,641/3134
*R*_factor_/*R*_free_ (%) ^1,3^	19.0/25.7	10.0/23.9
No. of atoms (Protein/Ligand/Water)	7506/-/167	7621/86/280
**Model Quality**		
R.m.s deviations		
Bond lengths (Å)	0.010	0.009
Bond angles (°)	0.969	0.906
Mean B-factor (Å^2^)		
All Atoms	53.9	46.0
Protein	54.1	45.9
Ligand	-	63.1
Water	45.4	42.8
Coordinate error (maximum likelihood) (Å)	0.33	0.29
Ramachandran Plot		
Most favored (%)	94.4	95.9
Additionally allowed (%)	4.9	3.6

^1^ Values in parenthesis are for the highest resolution shell. ^2^ *R*_merge_ = Σ*_hkl_*Σ*_i_* |*I_i_*(*hkl*) − <*I*(*hkl*)>|/Σ*_hkl_*Σ*_i_ I_i_*(*hkl*), where *I_i_*(*hkl*) is the intensity measured for the *i*th reflection and <*I*(*hkl*)> is the average intensity of all reflections with indices *hkl*. ^3^ *R*_factor_ = Σ*_hkl_* ||*F*_obs_ (*hkl*) | − |*F*_calc_ (*hkl*) ||/Σ*_hkl_* |*F*_obs_ (*hkl*)|; R_free_ is calculated in an identical manner using 5% of randomly selected reflections that were not included in the refinement. ^4^ *R*_meas_ = redundancy-independent (multiplicity-weighted) *R*_merge_ [28,29]. *R*_pim_ = precision-indicating (multiplicity-weighted) *R*_merge_ [30,31]. ^5^ CC_1/2_ is the correlation coefficient of the mean intensities between two random half-sets of data [32,33].

## Data Availability

Crystallographic structures have been submitted to the PDB.

## Supplementary Materials

The following supporting information can be downloaded at: https://www.mdpi.com/article/10.3390/toxins15070410/s1, Table S1: pK_a_ values of the titratable residues in the diphtheria toxin T-domain calculated using web-based H^++^ tools from X-ray structures reported here and in Ref. [34]. Reference [67] is cited in the supplementary materials.
