# Supplementary material for: Histidine Protonation and Conformational Switching in Diphtheria Toxin Translocation Domain
Source: Toxins (Basel). 2023 Jun 25;15(7):410. doi: 10.3390/toxins15070410 (PMC10467104; doi:10.3390/toxins15070410)
Supplement: Supplementary file 1 [file toxins-15-00410-s001.zip › toxins-2396824-supplementary.pdf]

Supplementary Materials for:

Histidine Protonation and Conformational Switching in Diphtheria Toxin Translocation Domain

Mykola V. Rodnin[1], Victor Vasques-Montes[1], Alexander Kyrychenko[1,2], Nuno F.B. Oliveira[3], Maithri M. Kashipathy[4], Kevin P. Battaile[5], Justin Douglas[6], Scott Lovell[4], Miguel Machuqueiro[3] and Alexey S. Ladokhin[1]*

[1]University of Kansas School of Medicine, Department of Biochemistry and Molecular Biology, Kansas City, KS, USA

[2] Institute of Chemistry and School of Chemistry, V. N. Karazin Kharkiv National University, Kharkiv 61022, Ukraine

[3] Institute of Biosystems and Integrative Sciences, University of Lisbon, Lisbon, Portugal

[4]Protein Structure and X-ray Crystallography Laboratory, University of Kansas, Lawrence, KS, USA

[5] NYX, New York Structural Biology Center, Upton, NY, USA

[6]COBRE Bio-NMR Laboratory, University of Kansas, Lawrence, KS, USA

Phone: 913-588-0489 FAX: 913-588-7440 e-mail: aladokhin@kumc.edu

Table S1. pKa values of the titratable residues in the diphtheria toxin T-domain calculated using web-based H++ tools from X-ray structures reported here and in [2].

Residue	WT (PDB 7K7E pH 7.0) [2]	WT (PDB 7K7C pH 5.5) [2]	H223Q/H257Q (PDB 8G0F pH 5.5)
Asp 205	3.2	3.2	3.2
Asp 207	4.0	4.0	3.9
Asp 211	4.1	3.9	3.7
Glu 218	4.0	4.0	4.2
Glu 222	3.7	3.7	4.2
**His 223**	**4.6**	*Not resolved*	**-**
Glu 232	4.1	4.0	3.3
Glu 240	2.3	2.8	3.4
Glu 241	4.0	3.9	4.1
Glu 248	4.1	4.0	4.6
Glu 249	3.6	3.6	2.9
**His 251**	**5.5**	**5.6**	**6.1**
Glu 256	3.4	3.2	3.9
**His 257**	**2.5**	**4.3**	**-**
Glu 259	1.9	1.8	4.0
Glu 262	3.5	4.0	4.0
Asp 290	3.4	3.3	3.2
Glu 292	4.2	4.3	4.3
Asp 295	4.0	3.9	3.8
Glu 298	4.2	4.2	4.2
Asp 318	3.3	3.1	3.4
**His 322**	**6.7**	**6.7**	**6.9**
**His 323**	**4.0**	**4.5**	**2.9**
Glu 326	2.8	2.7	3.2
Glu 327	2.0	1.1	0.8
Glu 349	5.1	5.2	5.0
Asp 352	2.8	2.8	2.9
Glu 362	2.7	2.7	3.0
**His 372**	**3.1**	**3.3**	**3.5**

References:

1. Gordon, J.C.; Myers, J.B.; Folta, T.; Shoja, V.; Heath, L.S.; Onufriev, A. H++: a server for estimating pKa and adding missing hydrogens to macromolecules. *Nucleic Acids Res.* **2005**, *33*, W368-W371, doi:10.1093/nar/gki464.
2. Rodnin, M.V.; Kashipathy, M.M.; Kyrychenko, A.; Battaile, K.P.; Lovell, S.; Ladokhin, A.S. Structure of the Diphtheria Toxin at Acidic pH: Implications for the Conformational Switching of the Translocation Domain. *Toxins* **2020**, *12*, 704, doi:10.3390/toxins12110704.
